# Triple stimulation (TriStim) before bilateral oophorectomy in a young woman with ovarian cancer: a case report and review of the literature

**DOI:** 10.1186/s40738-020-00087-y

**Published:** 2020-10-26

**Authors:** Laura C. Gemmell, Jason D. Wright, Paula C. Brady

**Affiliations:** 1 Department of Obstetrics and Gynecology, Columbia University Irving Medical Center/New York Presbyterian Hospital, 622 west 168th street, New York, NY 10032 USA; 2 Department of Obstetrics and Gynecology, Division of Gynecologic Oncology, Columbia University Irving Medical Center, New York, USA; 3 Department of Obstetrics and Gynecology, Columbia University Fertility Center, Columbia University Irving Medical Center, New York, USA

**Keywords:** Double ovarian stimulation, DuoStim, Fertility preservation, Oncofertility, Triple stimulation, TriStim

## Abstract

**Background:**

Double ovarian stimulation (DuoStim) involves two rounds of controlled ovarian stimulation (COS) and oocyte retrieval in immediate succession. It represents a promising approach to increase oocyte yield for patients with diminished ovarian reserve or those with limited time before fertility-threatening oncologic treatment. We report the case of a 31-year-old woman with Stage IC endometrioid ovarian cancer who underwent a triple stimulation or “TriStim,” completing three rounds of COS and oocyte retrieval within 42 days prior to bilateral salpingo-oophorectomy.

**Case presentation:**

A 31 year old nulligravid woman presented for fertility preservation counseling following a bilateral ovarian cystectomy that revealed Stage IC endometroid adenocarcinoma arising within endometrioid borderline tumors. The patient was counseled for bilateral salpingo-oophorectomy, lymph node dissection, and omentectomy followed by three cycles of carboplatin/paclitaxel. Prior to this, all within six weeks, the patient underwent three rounds of controlled ovarian stimulation using an antagonist protocol and human chorionic gonadotropin (hCG) trigger, resulting in vitrification of nine two-pronuclear zygotes (2PN), after which definitive surgery was performed.

**Conclusions:**

Advantages of DuoStim procedures are increasingly recognized, especially for oncology patients with limited time before potentially sterilizing cancer treatment. To our knowledge, this is the first report of a triple stimulation (“TriStim”). Our case highlights that triple stimulation is a viable option for patients needing urgent fertility preservation in order to maximize egg and embryo yield within a limited time period.

## Background

Traditional teaching of follicular development focuses on a single cohort of antral follicles that grow in response to gonadotropins in the early follicular phase of the menstrual cycle. As a result, conventional ovarian stimulation begins on cycle day two or three. With advances in transvaginal ultrasound, studies have challenged the traditional paradigm by suggesting that women experience waves of follicular growth during the interovulatory interval [1].

Waves of folliculogenesis allow for “random start” and double ovarian stimulation protocols. “Random start” refers to ovarian stimulation initiated at any time during the menstrual cycle, ideal for patients with limited time for assisted reproductive technologies [2]. Double ovarian stimulation or “DuoStim” consists of a cycle of controlled ovarian stimulation and oocyte retrieval immediately following a prior stimulation and retrieval without waiting for progesterone to return to baseline. The second stimulation starts in the luteal phase, which appears to result in a higher oocyte yield and similar embryo euploidy rate for patients with diminished ovarian reserve [3–6]. This increase reflects not only the doubled number of retrievals, but also the higher oocyte yield of the luteal phase second start [4, 7]. DuoStim also increases the number of mature oocytes cryopreserved for oncology patients [8].

To our knowledge, this is the first report of triple stimulation (“TriStim”), meaning three controlled ovarian stimulation protocols in immediate succession. We report the case of a 31-year-old woman with ovarian cancer who underwent a successful triple stimulation prior to bilateral oophorectomy.

## Case presentation

We report the case of a 31-year-old nulliparous woman diagnosed with stage 1C ovarian cancer in 2019. She initially presented with bilateral complex ovarian cysts and a significantly elevated cancer antigen 125 (CA-125) of 526 U/mL. She underwent bilateral laparoscopic ovarian cystectomies with pathology significant for endometroid ovarian carcinoma arising in bilateral endometrioid borderline tumors. Her medical history was unremarkable except for daily tobacco use (two cigarettes per day). The patient was counseled for bilateral salpingo-oophorectomy, lymphadenectomy, and omentectomy followed by three cycles of carboplatin/paclitaxel. Testing for Lynch Syndrome was pending at the time of consultation for fertility preservation, but eventually returned negative.

Evaluation revealed an anti-mullerian hormone (AMH) level of 0.39 ng/mL and an antral follicle count of 3 by ultrasound, along with moderate free fluid due to recent bilateral ovarian cystectomy. Her 34-year-old partner’s semen analysis was poor with a low concentration (4.7 million/mL), low motility (16%), and no morphologically normal sperm (0%). He was referred to urology for further evaluation, which is ongoing.

She then underwent three cycles of controlled ovarian stimulation spanning 42 days. Her first cycle began just 3 weeks after bilateral ovarian cystectomy as a random start in the luteal phase; a gonadotropin-releasing hormone antagonist (GnRH-antagonist) protocol was utilized with 450 units of gonadotropins per day [9]. Letrozole (aromatase antagonist, 5 mg) was prescribed due to theoretical concern for estrogen receptors in her gynecologic cancer, but was discontinued after five days due to side effects. She was stimulated for eleven days, reached a peak estradiol (E2) of 261 pg/mL, and triggered with human chorionic gonadotropin (hCG) once two lead follicles reached a diameter of at least 18 mm. One metaphase II (MII) oocyte was retrieved, fertilized by intracytoplasmic sperm injection (ICSI), and frozen at the two pro-nuclear (2PN) stage.

Her second cycle with the same protocol was initiated six days after retrieval; following nine days of stimulation and a peak E2 of 2072 pg/mL, eight oocytes (6 MII) were retrieved, and three fertilized and were frozen at the 2PN stage. An immature oocyte (MI) underwent in vitro maturation (IVM) overnight, fertilized, and was frozen as a 2PN. Immature oocytes that did not respond to in vitro maturation were not cryopreserved. Her third cycle was initiated six days after her second retrieval. After ten days of stimulation with a peak estradiol of 1543 pg/mL, seven oocytes (6 MII) were retrieved with four fertilized and frozen as 2PNs. The six day interval between stimulations was due to the patient’s schedule, including other medical appointments.

With multiple embryos cryopreserved, she underwent a laparoscopic bilateral salpingo-oophorectomy, lymph node dissection, and omentectomy with pathology significant for a small residual focus of endometrioid borderline tumor. The patient declined chemotherapy and is being monitored with frequent laboratory testing and imaging. She has been advised by her gynecologic oncologist to wait at least one year after definitive surgery prior to embryo transfer.

## Discussion and conclusions

One in 18 women will be diagnosed with cancer before age 50, and cancer survivors are now living longer due to advances in therapies [10, 11]. However, many cancer treatments, including alkylating chemotherapy agents and pelvic radiation, may negatively affect ovarian reserve or induce premature menopause [12]. Patients with ovarian cancer are typically managed with total hysterectomy and bilateral salpingo-oophorectomy, however, fertility sparing surgery may be possible dependent on the histologic subtype and stage of disease. Reproductive risks of cancer treatment should be discussed with all reproductive age patients, who should be referred to a reproductive endocrinologist to review options, including oocyte, embryo, or ovarian tissue cryopreservation in women [13]. Ovarian cancer patients are candidates for oocyte or embryo cryopreservation, however, ovarian tissue cryopreservation is discouraged given the theoretical risk of cancer recurrence upon reimplantation [14]. Oncology treatment often must be initiated urgently, leaving limited time for fertility preservation. Particularly for patients with limited time for treatment, random start protocols are a more convenient and equally effective alternative to conventional follicular-start ovarian stimulation [15–17].

In recent years, the principles of random and luteal starts have been taken a step further with the DuoStim, involving two controlled ovarian stimulations in rapid succession without waiting for progesterone to return to baseline, in order to maximize the number of oocytes retrieved without delaying cancer treatment [8]. The optimal timing to wait between stimulations is currently unknown, although second stimulation cycles beginning as early as the day of first oocyte retrieval have been published [18]. DuoStim maximizes oocyte yield not only by increasing the number of cycles, but also due to the higher yield of luteal phase starts [4, 7]. Proposed mechanisms for increased oocyte yield in the luteal phase stimulation include synchronization of antral follicles due to high estrogen and progesterone during follicular stimulation and increased angiogenic factors following follicular stimulation leading to enhanced granulosa cell sensitivity to follicle stimulating hormone (FSH). Additionally, in follicular stimulations completed with a GnRH agonist trigger, the flare effect may downregulate AMH expression in follicles leading to improved antral follicle recruitment [4].

To our knowledge, no triple stimulation or “TriStim” protocols have previously been published. Our patient underwent three cycles of controlled ovarian stimulation and egg retrieval over a total of 42 days, resulting in 16 oocytes and 9 2PN embryos, an excellent outcome in the context of a low starting AMH. While DuoStim protocols typically utilize a GnRH agonist for final oocyte maturation in cancer patients to decrease the risk of ovarian hyperstimulation syndrome and further delays in care, hCG was selected due to cost and the patient’s low risk of ovarian hyperstimulation as judged by AMH and stimulation response. Embryos were frozen at the 2PN stage as testing for cancer susceptibility genes was still pending at that point and the need for preimplantation genetic testing was unknown. Additionally, although ICSI was used in this case due to poor semen parameters, the use of ICSI may also be preferred in patients for whom preimplantation genetic testing for monogenic disorders (PGT-M) may be necessary in the future. At this point, the patient’s Lynch syndrome testing was still pending.

The lower response in her first cycle may have been due to the closer proximity to her bilateral ovarian cystectomy (3 weeks prior), and/or the utilization of letrozole, which has been variably correlated with reduced oocyte yield in oncology patients [19]. Letrozole, an aromatase inhibitor, has been used in controlled ovarian stimulation protocols in breast cancer patients in an attempt to decrease supraphysiologic serum estrogen levels that may stimulate estrogen receptor positive tumor cells [20]. Letrozole was used initially due to concern for estrogen receptor expression in endometrioid tumors [21]. However, there is no high quality data demonstrating the superiority of concomitant letrozole in gynecologic cancer patients [22]. The patient presented in this case report ultimately discontinued letrozole due to side effects.

This case demonstrates that women with limited time for controlled ovarian stimulation may benefit from triple stimulation if time allows, consistent with prior studies demonstrating the utility of DuoStim in both patients with diminished ovarian reserve and oncologic patients. This approach demonstrates that ongoing waves of folliculogenesis can be successfully harnessed in sequential rounds of ovarian stimulation, in preparation for (but without delaying) gonadotoxic treatment or gonadectomy. Additionally, given the higher oocyte yield observed in subsequent stimulations, this approach may be applicable to a patient population beyond oncology patients. Further research is warranted, although patients with other time pressures including HLA matching for a sick child, infertility patients with diminished ovarian reserve, or even elective oocyte freezing patients may be potential candidates.

## Data Availability

N/A

## Abbreviations

2PN: two-pronuclear
AMH: anti-mullerian hormone
CA-125: cancer antigen 125
COS: controlled ovarian stimulation
DuoStim: double ovarian stimulation
TriStim: triple stimulation
E2: estradiol
GnRH: gonadotropin-releasing hormone
FSH: follicle stimulating hormone
hCG: human chorionic gonadotropin
ICSI: intracytoplasmic sperm injection
IVM: in vitro maturation
MI: metaphase I
MII: metaphase II
